# Annotations

**Published:** 1906-09-15

**Authors:** 


### ANNOTATIONS.

### Neovitalism in Physiology.

Professor Gotch in his able address on Physiology at the York meeting of the British Association enters a spirited and very necessary protest against the use of the term vital to explain physiological processes. Vitalism connotes no more in physiology than the term living, and its employment does not in any way enlarge our knowledge of the subject-matter of physiology. It is therefore either a meaningless tautology, if it be used in this sense, or it is an expression of faith which is quite inadmissible in the study of science. The term is sometimes used to convey some mystical, occult, or additional influence, and in this sense it is not only a cloak to our ignorance, but is actually detrimental to the progress of knowledge, because it offers a barrier to farther investigation. Vital action and calidum innatum were as dear to the older physiologists as were special creations to the older palæontologists. Calidum innatum disappeared with the progress of chemistry and physics. The vital principle has been driven to explain more and more recondite processes as the simpler problems have proved capable of explanation by ordinary scientific means. Every year the pioneers in the different branches of physiology feel themselves less called upon to invoke vitalism as an explanation, and although for many years to come there will be much that cannot be explained in the actions of life, it is better to wait or to obtain a scientific explanation than to baffle investigation by the use of a purely philosophical expression which conveys no rational meaning.

### Doctors Aboard Ship.

The commonly recommended sea voyage for the benefit of health has one great disadvantage, inasmuch as the invalid aboard ship can never count on securing the services of an experienced seagoing practitioner. It is the common practice of steamship companies to engage newly qualified youths at a ridiculously low salary, who are anxious for free travel round the world to mend their broken health after the arduous labours of student life and the unkindnesses of hardened examiners. Sometimes a man is appointed whose health has threatened to give way, and whose friends think he will be benefited by a sea voyage. Others to whom our sympathies freely extend are compelled through stress of *res angusta domi* to accept such invidious positions as a means of earning a livelihood—a scanty livelihood at the best—often obtained not only at great physical disadvantage, but with an irreparable loss of professional dignity. The emoluments amount to £10 per month or thereabouts, with access to the saloon at meal times, and the use of a cabin to sleep in. We have known the doctor in one vessel belonging to a well-known company, actually having his quarters and state cabin forward among the crew. Even in vessels where accommodation for the doctor is of a higher character, the company sub-let the services of the practitioner as part of the value they give in return for passage money. This is unfair, not only to the medical officer, but to the passengers, and it is highly improper that a doctor, a gentleman and a scholar, a member of a profession in which dignity and good-feeling is a part of its stock-in-trade, should be compelled to look for an augmentation of his income by way of tips or gratuities much in the same way as if he were a bedroom steward or a pantry boy. Gentlemen must be humiliated by this process, and many patients are prevented from taking advantage of a medical man's services and advice on this account. Steamship companies doubtless know their own business best, but we think it would be to their interest as well as to the interest of the public if things medical on board ship were ordered differently.

### Kissing the Book.

On more than one occasion in this column we have directed attention to the disagreeable and insanitary method of administering the oath commonly, indeed universally, adopted in the English courts of justice. Hence we are more than ready to respond to the protest recently made by Sir Thomas Snagge in his capacity as judge of the county court at Northampton. In response to an objection to "kiss the book" advanced by a witness, Sir Thomas energetically condemned the prevalent practice as an unhealthy, insanitary, and objectionable formality, and expressed a decided opinion that it ought to be abolished. He, however, went further than this, and appealed to the medical profession for support. By so doing the judge showed what may be termed an accurate appreciation of the method by which the desired reform is likely to be accomplished. So far as can be judged from reading and conversation, no one has a word to say in defence of the present practice, and many profess strong objections to it. Yet such is the force and sway of custom that even those who dislike the existing method most strongly, submit quietly rather than run the risk of criticism at a trying and conspicuous moment. Some decided and sustained object-lesson is evidently necessary to assure the public that it is open to any witness who so desires it to be sworn in the Scottish fashion, that is, by repeating an oath with uplifted hand instead of kissing the book. All that is necessary is for the witness to make the request. Neither the judge nor anyone else is entitled to object or to question the witness on the subject. Now if every medical man would adopt this attitude can it be doubted that the public would promptly follow the example? Notices in court, intimations from the bench, articles in the Press, have produced but little effect in modifying a nauseous and unhealthy practice. The motive force needed is that of example. And when the question is one of cleanliness and sanitary prudence the medical profession surely ought to lead the way. We have repeatedly urged this, and we are therefore pleased to have our argument supported by an appeal from the judicial bench.

